# Preparation and High-Temperature Resistance Properties of Phenolic Resin/Phosphate Hybrid Coatings

**DOI:** 10.3390/ma17092081

**Published:** 2024-04-28

**Authors:** Qinzhe Li, Yu Zhang, Lizhen Zhou, Peng Lei, Jiangyan Liu, Fuli Wang, Xueyun Xiang, Hang Wu, Wen Wang, Fuhui Wang

**Affiliations:** 1 Shenyang National Laboratory for Materials Science, Northeastern University, Shenyang 110819, China; 13205489190@163.com (Q.L.); wszb157@163.com (Y.Z.); lizhen20000425@163.com (L.Z.); leipeng1999106@163.com (P.L.); ljy081722@163.com (J.L.); wangfuli1122@163.com (F.W.); xiangxueyun1998@163.com (X.X.); wen@imr.ac.cn (W.W.); fhwang@mail.neu.edu.cn (F.W.); 2 School of Materials Science and Engineering, Northeastern University, Shenyang 110819, China; 3 Institute of Metals, Chinese Academy of Sciences, Shenyang 110016, China

**Keywords:** organo–inorganic hybrid coatings, phenolic resin, phosphate, antioxidative behavior, porosity

## Abstract

In this study, a novel fabrication method was used to synthesize phenolic resin/phosphate hybrid coatings using aluminum dihydrogen phosphate (Al(H_2_PO_4_)_3_, hereafter denoted as Al), SC101 silica sol (Si) as the primary film-forming agent, and phenolic resin (PF) as the organic matrix. This approach culminated in the formation of Al+Si+PF organo–inorganic hybrid coatings. Fourier-transform infrared spectroscopy (FT-IR) and X-ray photoelectron spectroscopy (XPS) results confirmed the successful integration of hybrid structures within these coatings. The crystalline structure of the coatings post-cured at various temperatures was elucidated using X-ray diffraction (XRD). Additionally, the surface and cross-sectional morphologies were meticulously analyzed using scanning electron microscopy (SEM), offering insights into the microstructural properties of the coatings. The coatings’ porosities under diverse thermal and temporal regimes were quantitatively evaluated using advanced image processing techniques, revealing a significant reduction in porosity to a minimum of 5.88% following a thermal oxidation process at 600 °C for 10 h. The antioxidant efficacy of the phosphate coatings was rigorously assessed through cyclic oxidation tests, which revealed their outstanding performance. Specifically, at 300 °C across 300 h of cyclic oxidation, the weight losses recorded for phosphate varnish and the phenolic resin-infused phosphate coatings were 0.15 mg·cm^−2^ and 0.09 mg·cm^−2^, respectively. Furthermore, at 600 °C and over an identical period, the weight reduction was noted as 0.21 mg·cm^−2^ for phosphate varnish and 0.085 mg·cm^−2^ for the hybrid coatings, thereby substantiating the superior antioxidation capabilities of the phenolic resin hybrid coatings in comparison to the pure phosphate varnish.

## 1. Introduction

In contemporary China, materials employed across various critical sectors, such as metallurgy, chemical industry, military, and energy, face formidable challenges stemming from high-temperature oxidation. In environments characterized by prolonged exposure to high temperatures and oxygen, metallic materials encounter significant risks to their performance and structural integrity. Specifically, metallic components found in petroleum cracking apparatuses, high-temperature conveyance systems, and boilers in power stations exhibit increased vulnerability to oxidative reactions in the presence of oxygen [1,2]. These reactions not only accelerate the aging process of the materials but also pose potential risks for safety incidents.

In response to these challenges, phosphate coatings represent a pivotal advancement in the inorganic coating technologies for metal surfaces. Owing to their remarkable adhesive and sealing characteristics, these coatings have been extensively used in high-temperature environments. Phosphate coatings are highly effective in bolstering the resistance of materials against high-temperature oxidation. By establishing a dense and impervious layer, these coatings act as a formidable barrier that shields the material from oxygen, moisture, and a wide array of chemical agents. This protective layer plays a crucial role in preserving the integrity of the underlying metal, offering substantial defense against corrosion. Consequently, phosphate coatings are indispensable for enhancing the durability and longevity of metal surfaces, ensuring they remain protected even in the most challenging environments. Phosphate coatings can significantly enhance the resistance of materials to high-temperature oxidation by forming an impermeable barrier against oxygen, moisture, and various chemical agents, which, in turn, provides a robust safeguard for the underlying metal against corrosion, thereby playing an essential protective role [3,4,5,6,7].

Extensive research has been conducted globally in recent years to augment the high-temperature resilience of phosphate coatings. Han et al. [8] innovatively crafted phosphate coatings on a Ti65 substrate using Al(NO_3_)_3_ and P_2_O_5_, leading to the formation of AlPO_4_ as the principal film-forming agent. These layers, meticulously applied through air spraying techniques to establish aluminum phosphate layers, exhibited exceptional adhesion to the substrate, maintaining structural integrity without significant surface cracking even after prolonged exposure to oxidation at 650 °C. This process substantially improves the antioxidative capabilities of the Ti65 substrate. Huang et al. [9] explored the use of Al(H_2_PO_4_)_3_ as the primary film-forming agent, Al_2_O_3_ as a hardening agent, and nano-CeO_2_ as a modifier for coatings. Utilizing a vapor-assisted deposition method, they created aluminum phosphate coatings that adhered optimally to an Al-Si-cast aluminum alloy substrate enhanced with 2 wt.% CeO_2_. Adding nano-CeO_2_, known for its high chemical reactivity within the inorganic phosphate matrix, was instrumental in impeding the diffusion of detrimental ions, thereby postponing the corrosion of the substrate and facilitating the curing of the phosphate coating at temperatures below 200 °C. Similarly, Liu et al. [10] advanced the field by incorporating tetrapod-like ZnO whiskers coated with alumina (T-ZnOw@Al_2_O_3_) into the phosphate matrix, yielding a coating that preserved its surface roughness and antioxidation efficiency and performed admirably at temperatures as high as 800 °C. These studies collectively highlight the dynamic advancements in enhancing the high-temperature resistance of phosphate coatings through strategic material selection and processing techniques.

In contemporary research on phosphate coating formulations, the majority of studies are predominantly focused on evaluating the high-temperature oxidation resistance of inorganic fillers within these coatings. However, investigations involving organic fillers hybridized with inorganic phosphate coatings are notably less frequent. Compared to their purely inorganic counterparts, coatings hybridized with organic phenolic resins exhibit marked improvements in reducing brittleness and porosity, thereby enhancing their flexibility during both preparation and application processes. These advancements address significant limitations inherent to purely inorganic phosphate coatings.

One promising strategy to mitigate these drawbacks involves organic hybridization of the phosphate coating framework. We have tested three different types of organic resins as fillers for composite coatings: organosilicon, waterborne epoxy emulsion, and phenolic resin. The results indicate that organosilicon does not mix well with the phosphate system, leading to layer separation; the waterborne epoxy emulsion exhibits a low carbonization rate at high temperatures, significantly increasing the porosity of the composite coating. In contrast, the phenolic resin integrates well with the system, exhibiting higher carbonization yield at elevated temperatures, thereby enhancing the density and flexibility of the coating. Therefore, phenolic resin is ultimately chosen as the organic filler. Among the several available thermosetting resins, phenolic resins are notable for their superior thermal resistance, insulation properties, enhanced flexibility, and notable char yields. Considering these advantageous properties, the current study used a phenolic resin to create an innovative organo–inorganic hybrid coating by amalgamating it with inorganic phosphate coatings. This study meticulously evaluated the cyclic oxidation resistances of these novel coatings, both before and after hybridization, under various high-temperature scenarios. The evaluation techniques employed included scanning electron microscopy (SEM) for microstructural analysis, Fourier-transform infrared spectroscopy (FT-IR) for chemical characterization, X-ray diffraction (XRD) for phase analysis, and porosity measurements after exposure to high-temperature conditions, thereby comprehensively assessing the enhanced performance and durability of the hybrid coatings.

## 2. Materials and Methods

### 2.1. Selection of the Substrate

The metal substrate selected for this study was 304 stainless steel, with its specific elemental composition presented in Table 1.

### 2.2. Chemical Reagents Utilized in the Experiment

The chemical reagents used in this study are listed in Table 2.

### 2.3. Preparation of the Substrate

Grade 304 stainless steel plates with dimensions of 20 × 20 cm and a thickness of 2 mm were cut into 20 × 20 mm specimens using a spark wire cutting machine. The specimens were sequentially polished with 400- and 800-grit SiC sandpaper to remove the oxide layer from the metal substrate surface and chamfer the edges. Subsequently, the polished specimens were subjected to abrasive blasting with 80-grit white corundum under a pressure of 0.4 MPa to enhance adhesion between the coating and substrate. Finally, the specimens were ultrasonically treated with an acetone and ethanol mixture for 10 min. After removal, the samples were air-dried and prepared for further use.

### 2.4. Preparation of the Coating

Phenolic resin/phosphate organo–inorganic hybrid coatings were prepared using the sol-gel method. Al(H_2_PO_4_)_3_ and SC101 silica sol were used as the primary film-forming substances, with phenolic resin serving as the filler. Initially, 30 g of SC101 silica sol, 60 g of Al(H_2_PO_4_)_3_, and an appropriate amount of ZrSiO_4_ grinding beads, which acted as the milling medium, were added to the flask. The mixture was agitated vigorously at a high speed of 2500 revolutions per minute (rpm) under ambient conditions for a duration of 30 min. In the formulation process of the paint, the ambient temperature was initially recorded at 25 °C before the commencement of stirring. During the stirring activity, the temperature slightly rose to 27.5 °C. This minor temperature increase, attributable to the thermal dissipation by the water component in the aqueous system, is considered negligible and thus does not significantly affect the experimental results. This process continued until the varnish transitioned to a milky white appearance. Subsequently, 10 g of phenolic resin was slowly added, and the mixture was stirred at a lower speed (1500 rpm) for 2 h to ensure thorough hybridization. During the stirring process, 0.5 g of the BYK-022 defoaming agent and 1.8 g of the FS-50 wetting and dispersing agent were incorporated to ensure the wettability of the coating for subsequent spraying. The final coating was obtained by filtering through a 200-mesh screen. The prepared coating was loaded into a W-71 type spray gun with a spraying pressure of 0.2 MPa and applied evenly to the surface of 304 stainless steel using ambient air spraying. After each coating application, the samples were air-dried for 30 min. A second coating layer was applied and allowed to air dry for 2 h, followed by forced-air drying and curing at 60 °C for 8 h. Upon cooling, phenolic resin/phosphate hybrid coating samples were successfully formed on the substrate surface.

### 2.5. Characterization of Coating Performance

#### 2.5.1. Fourier Transform Infrared Spectroscopy Analysis (FT-IR)

The chemical structures and functional groups of the coatings were characterized using a Nicolet iS20 Fourier-transform infrared spectrometer (Thermo Scientific, Waltham, MA, USA). The spectrometer was operated with a resolution of 4 cm^−1^ and 64 scans were conducted across a measurement range of 4000–400 cm^−1^. To prepare the samples for analysis, the coatings were ground into powder and subjected to the potassium bromide (KBr) pellet method for sample preparation.

#### 2.5.2. X-ray Photoelectron Spectroscopy Analysis (XPS)

XPS analysis was employed for qualitative and quantitative elemental analyses of the phosphate coatings before and after hybridization. A Kratos-Axis Supra X-ray Photoelectron Spectrometer (San Diego, CA, USA) was used to investigate the chemical composition and state of the coating surfaces. Charge compensation in the XPS spectra was achieved using the C1s peak at 284.8 eV, corresponding to the C–C single bond, with the Al Kα line serving as the X-ray source.

#### 2.5.3. X-ray Diffraction Analysis (XRD)

Structural analyses of the phosphate coatings before and after hybridization were conducted using a SmartLab X-ray Diffractometer (San Francisco, CA, USA) with a cobalt target as the radiation source. The analysis covered a range of 5–80°, with the target rotating at a speed of 5°/min and a step size of 0.01°.

#### 2.5.4. Scanning Electron Microscopy Analysis (SEM)

The surface and cross-sectional microstructures of the experimental samples were observed at various stages using an Inspect F50 Scanning Electron Microscopy (Hillsboro, OR, USA) with the tests performed at an accelerating voltage of 15 kV. Before the morphological characterization, the coatings were sputter-coated with gold to enhance their electrical conductivity and improve the image quality.

#### 2.5.5. High-Temperature Oxidation Analysis

The high-temperature oxidation experiments were conducted in a custom-designed tubular furnace, with the samples subjected to oxidation at temperatures of 300 °C, 600 °C, and 900 °C for 1 h, 10 h, and 100 h, respectively. Following high-temperature oxidation, the microstructural morphology of the coatings was characterized to investigate the behavior of the phenolic resins under various conditions. The porosity of the post-oxidation coatings was quantitatively assessed using image processing software, enabling the determination of the porosity of the phenolic resin coatings across different temperatures and durations.

#### 2.5.6. Cyclic Oxidation Analysis

Cyclic oxidation analysis was conducted by subjecting the samples to static oxidation at temperatures of 300 °C and 600 °C for 60 min, followed by air cooling for 15 min; this process constituted one cycle. The mass of each sample was measured and recorded every 20 cycles to monitor changes in mass, with the experiment spanning 300 cycles for a total oxidation duration of 300 h. To ensure accuracy and reproducibility, three parallel samples were prepared for each specimen type, with the results expressed as the average mass change among these samples. A 304 alloy substrate was used as the baseline control to facilitate comparative analysis.

## 3. Results and Discussion

### 3.1. Characterization by Fourier Transform Infrared Spectroscopy (FT-IR)

**Figure 1:**
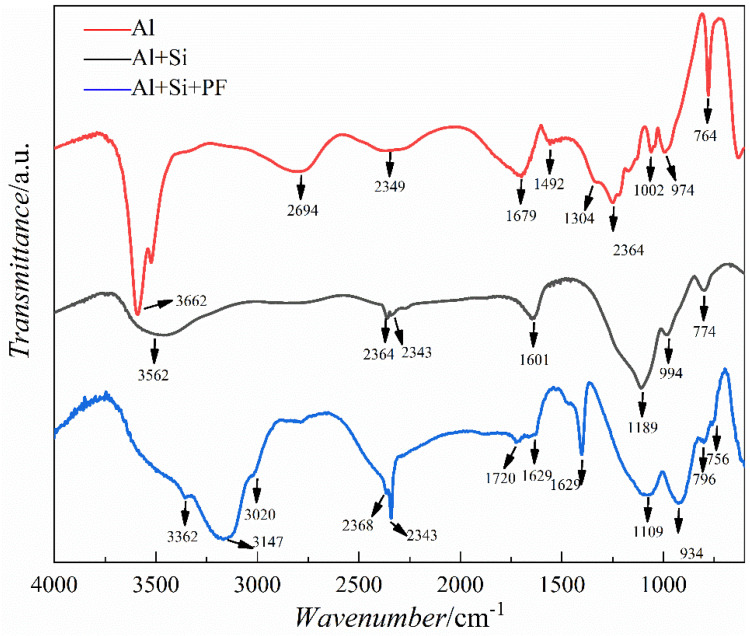
Infrared spectra of cured products of Al, Al+Si, and Al+Si+PF film formers.

In this study, FT-IR analyses were conducted on solid powders obtained from the curing at 60 °C for 48 h of the basic film-forming Al(H_2_PO_4_)_3_ (Al), a mixed solution of Al(H_2_PO_4_)_3_ and silica sol (Al+Si), and a composite solution of Al(H_2_PO_4_)_3_, silica sol (Si), and phenolic resin (PF) (Al+Si+PF). Figure 1 shows the FTIR spectra of the three formulations. The results indicate that the phenolic resin hybridized phosphate solution exhibits absorption peaks near 3362 cm^−1^, 3147 cm^−1^, 3020 cm^−1^, 2368 cm^−1^, 2343 cm^−1^, 1720 cm^−1^, 1629 cm^−1^, 1404 cm^−1^, 1109 cm^−1^, 934 cm^−1^, and 796 cm^−1^. These absorption peaks correspond to the stretching vibrations of different chemical bonds between Si, P, and O atoms and the vibrations of new chemical bonds arising from the hybridization of the phenolic resin with the phosphate solution. Based on existing research on chemical bonding within SiO_2_-P_2_O_5_-B_2_O_3_ glass systems and organics, the peak near 1100 cm^−1^ is indicative of the P=O [11] double bond vibration. Furthermore, the peaks appearing within the 900–1250 cm^−1^ range are representative of the asymmetric bending vibrations of the X-O (X = P/Si) bonds present within the tetrahedral [SiO_4_] and [PO_4_] structures in the cured products, consistent with the fundamental understanding of the molecular structure and dynamics within such glass and hybrid organic–inorganic systems, thus providing insight into the intricate bond interactions and structural conformations post-curing [12,13,14]. The peak observed near 1630 cm^−1^ is attributed to stretching and bending vibrations of the P-O bond. This spectral feature provides crucial insight into the dynamic interactions and structural integrity of the P-O linkage, reflecting the complex vibrational modes that contribute to the chemical properties of the material [15]. The peak at 3147 cm^−1^ corresponds to the stretching vibrations of the O-H bond in bound water, demonstrating the presence of hydration in the material. In the phosphate varnish formulation without phenolic resin hybridization (Al+Si), the absorption peaks at 994 cm^−1^ and 1189 cm^−1^ correspond to the asymmetric vibrations of the [Si-O-P] structure. The absorption peak near 2364 cm^−1^ is associated with the structural vibrations induced by the bridging of Al^3+^ ions between the [SiO_4_] and [PO_4_] units. This detailed spectroscopic analysis underscores the intricate molecular interactions and structural modifications within the phosphate varnish, revealing the pivotal role of aluminum ions in facilitating complex network formation between the silica and phosphate constituents [16,17,18]. Consequently, within the solution, Al(H_2_PO_4_)_3_ and silica sol undergo two key reactions, denoted as (1) [19,20] and (2) [21,22], which facilitate the formation of a hybrid structure. These reactions are instrumental in developing an intricate network that characterizes the hybrid material, leading to enhanced properties and performance owing to synergistic interactions between Al(H_2_PO_4_)_3_ and silica components.

P-OH + HO-Si → [-P-O-Si-] + H_2_O (1)

Si(OH)_4_ → [-Si-O-Si-] + H_2_O (2)

The film formation and curing processes are characterized by dehydration and condensation between adjacent hydroxyl groups. As the drying temperature increases, water evaporates in its gaseous form, facilitating the forward progression of the reaction. This mechanism underlies the self-sustaining nature of the film-forming reactions ((1) and (2)). Spectroscopically, the absorption peak near 1400 cm^−1^ corresponds to C–C stretching vibrations, a feature present in compounds containing aromatic rings. Moreover, the absorption peaks at 3362 cm^−1^ and 3020 cm^−1^ correspond to the stretching vibrations of the O-H and C-H bonds, respectively, in the hybrid organic phenol-formaldehyde compounds. These spectroscopic signatures provide molecular-level insights into the mechanisms governing the film formation and curing process [23]. To demonstrate that phenolic resin, an organic compound, has been thoroughly integrated into phosphate coatings, thereby completing the organic–inorganic hybridization process and producing a hybrid coating.

### 3.2. XPS Characterization of Phenolic Resin Hybridized Phosphate Coatings

**Figure 2:**
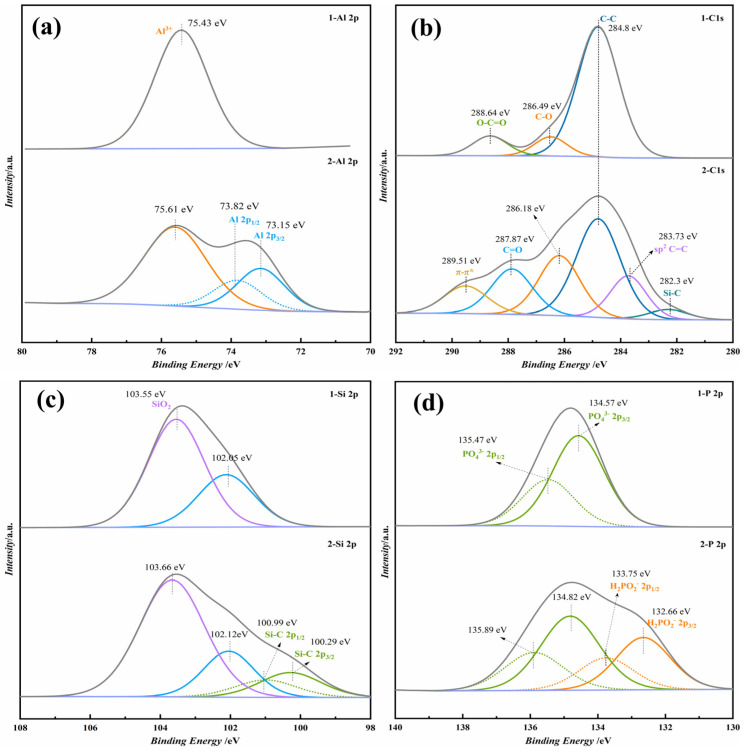
XPS map of phosphate coating before and after addition of phenol formaldehyde resin. (**a**) C 2p, (**b**) Si 2p, (**c**) P 2p, and (**d**) Al 2p fine spectra.

XPS analysis was performed to further verify whether the phenolic resin had been hybridized to the phosphate coating and to understand the impact of this hybridization on the surface structure of the coating, with the results presented in Figure 2. Sample 1 was a phosphate varnish without the hybridized phenolic resin, and Sample 2 was a phosphate coating hybridized with the phenolic resin. As depicted in Figure 2a, there was a discernible shift in the binding energy of carbon (C) following hybridization. The binding energy of the C-O bond shifts from 286.49 eV to 286.18 eV. Additionally, the spectrum after hybridization reveals the formation of new functional groups; the binding energy for the Si-C bond is 282.3 eV, alongside the appearance of sp^2^ hybridized C=C bonds and characteristic π-π* transitions of aromatic organic compounds, with their binding energies at 283.73 eV and 289.51 eV, respectively. Figure 2b demonstrates the shift in the binding energy of silicon (Si) after phenolic hybridization (from 103.55 eV to 103.66 eV) and the emergence of new chemical bonds Si-C in the 2p^1/2^ and Si-C 2p^3/2^ hybridized orbitals, with binding energies of 100.99 eV and 100.29 eV, respectively, indicating phenolic hybridization. Figure 2c,d show the binding energies for phosphorus (P) and aluminum (Al) elements, respectively. Compared to the non-hybridized phenolic resin, the hybridized P element exhibits the H_2_PO^2−^ valence state, with the H_2_PO^2−^ 2p^1/2^ and H_2_PO^2−^ 2p^3/2^ binding energies at 135.89 eV and 134.82 eV, respectively. The binding energy of Al shifts from 75.43 eV to 75.61 eV after phenolic hybridization, introducing new orbitals Al 2p^3/2^ and Al 2p^3/2^, with binding energies of 73.15 eV and 73.82 eV, respectively. The XPS results provided surface information on the phosphate hybrid coating, i.e., the phenolic resin was fully hybridized to the phosphate system, thus suggesting that the surface after hybridization comprised the reaction products of the phenolic resin, SiO_2_, Al(H_2_PO_4_)_3_, and others.

### 3.3. Phase Composition Analysis (XRD)

**Figure 3:**
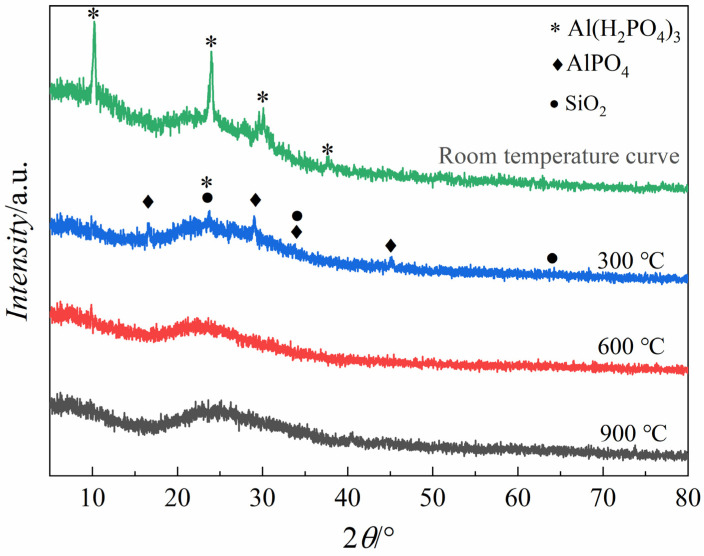
The X-ray diffraction (XRD) patterns of phosphate hybrid coatings that were cured at various temperatures: ambient conditions (room temperature), 300 °C, 600 °C, and 900 °C.

Investigating the thermal behavior and crystalline transformation mechanisms of phenol-formaldehyde hybridized with Al(H_2_PO_4_)_3_ revealed significant insights into material synthesis and applications in high-performance coatings. XRD analyses, as depicted in Figure 3, delineate the phase evolution of the synthesized composite powders subjected to calcination at temperatures of 300 °C, 600 °C, and 900 °C, respectively.

The initial room-temperature solidification of the hybrid material matrix led to the precipitation of Al(H_2_PO_4_)_3_ crystals, indicating incomplete dissolution of Al(H_2_PO_4_)_3_ within the hybrid coating matrix. Upon calcination at 300 °C for 1 h, the XRD patterns exhibited a noticeable suppression in crystal precipitation, leading to a reduction in Al(H_2_PO_4_)_3_ crystalline phases and the concurrent emergence of minor AlPO_4_ and SiO_2_ crystalline phases—indicative of the thermally induced transformation of Al(H_2_PO_4_)_3_ into AlPO_4_ under oxidative conditions facilitated by the high-temperature environment. The presence of [Si-O-P] and [Si-O-Si] structures within the matrix suggests the initiation of dehydration processes involving Si-O bonds, leading to the formation of SiO_2_ upon further thermal dehydration. At elevated temperatures of 600 °C and 900 °C, the composite powders exhibited a complete transition to an amorphous state, characterized by a broad hump between 20° and 30° in the XRD patterns, indicating the amorphous nature of the material. This transformation underscores the suitability of the synthesized phosphate hybrid coatings for applications under 600 °C and 900 °C conditions, thus highlighting the potential for advanced protective coatings in high-temperature applications.

### 3.4. Analysis of SEM Images

**Figure 4:**
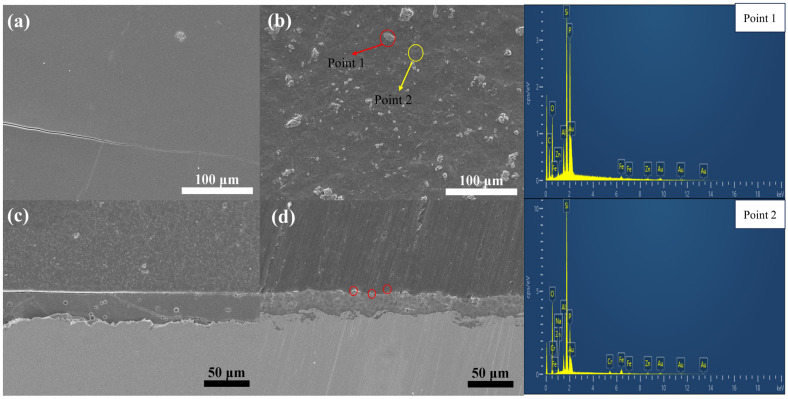
Microscopic morphology of phosphate varnish and hybrid coating. (**a**,**c**) show the cross-sections of the phosphate varnish, while (**b**,**d**) show the cross-sections of the phosphate hybrid coating.

To elevate this discourse to the caliber of top-tier scientific journals, we conducted an intricate analysis of the micro-morphology of phosphate varnish and phenol-formaldehyde resin/phosphate hybrid coatings after air drying at room temperature, followed by curing at 60 °C for 8 h, as illustrated in Figure 4.

Figure 4a,c show surface and cross-sectional micrographs of the phosphate varnish coating, respectively. As seen in Figure 4a, the surface of the coating contains numerous prominent cracks. Figure 4c further shows that, during the curing process, the delayed volatilization of some binding water leads to the formation of bubbles, thereby increasing the porosity and further diminishing the density of the coating. Moreover, cracks permeating from top to bottom in the cross-section of the phosphate varnish coating highlight the inherent brittleness of the coating, underscoring the necessity to enhance its flexibility and compactness. Figure 4b,d show the surface and cross-sectional micrographs of the water-soluble phenol-formaldehyde resin hybridized phosphate coating. As shown in Figure 4b, despite the phenol-formaldehyde resin being visibly dispersed throughout the hybrid coating (as per SEM results), it adopted an archipelago-like distribution on the surface, as indicated at Point 1. Incorporating the phenol-formaldehyde resin significantly enhances the compactness and flexibility of the coating. Comparatively, Figure 4d exhibits augmented compactness in the cross-section of the coating relative to Figure 4b, attributable to the role of the resin in mitigating through-and-through cracks, with the red circled area denoting a resin-rich region of organic phenol-formaldehyde. The red circled area in figure (d) represents the hybrid phenolic resin particles. The adjacent EDS images on the right further corroborate the successful hybridization of the phenol-formaldehyde resin. The elemental composition ratios of Points 1 (phenol-formaldehyde resin-hybridized phosphate coating) and 2 (phosphate varnish coating) are presented in Table 3 and Table 4, respectively.

This nuanced examination reveals the structural intricacies and challenges associated with the development of these coatings and highlights the enhancements achieved through the strategic incorporation of phenol-formaldehyde resin, steering the discourse toward potential advancements in coating technologies for high-performance applications.

**Figure 5:**
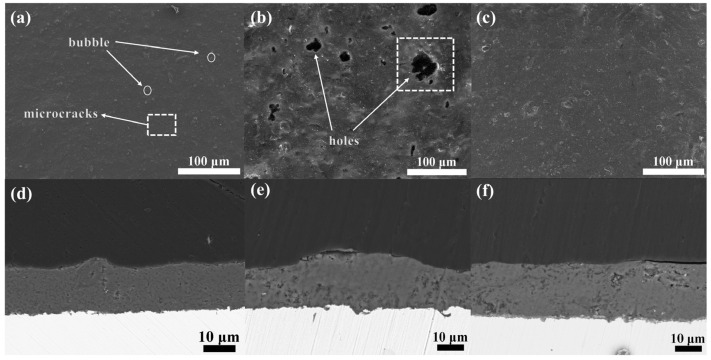
Surface and cross-sectional microstructure of phosphate coatings after high-temperature oxidation at 300 °C for 1 h, 10 h, and 100 h. (**a**,**d**) show the surface and cross-section of the coating after 1 h at high temperature; (**b**,**e**) show the surface and cross-section of the coating after 10 h at high temperature; (**c**,**f**) show the surface and cross-section of the coating after 100 h at high temperature.

Aligning with the stringent articulation and refinement expected in top-tier scientific journals, an advanced characterization of the surface and cross-sectional morphological features of hybrid phenol-formaldehyde resin phosphate coatings subjected to oxidative conditions at 300 °C for 1 h, 10 h, and 100 h is presented in Figure 5.

The micrographs depicted in Figure 5a,d indicate that, owing to the relatively short duration of high-temperature oxidation, the coating surface retained some fissures and vesicles, resulting in insufficient self-healing capability. Incorporating phenol-formaldehyde, even after a brief 1-h exposure, predominantly preserved its original morphology without substantial carbonization, as evidenced by the minimal transformation observed. In contrast, Figure 5b,e show the formation of pores on the coating surface after 10 h of high-temperature oxidation. This porosity arises from the phenol-formaldehyde resin undergoing carbonization, wherein the interaction with atmospheric oxygen leads to the volatilization of carbon dioxide, consequently compromising the compactness of the coating and increasing its porosity. Notably, Figure 5c,f demonstrate a significant enhancement in the self-healing capability after 100 h of oxidation, leading to a reduction in surface cracks. The prolonged carbonization of phenol formaldehyde contributed to the preservation of the carbon skeleton, which effectively inhibited the propagation of microcracks, thereby safeguarding the coating from extensive damage. This process improves the flexibility of the coating; moreover, owing to the high char yield of phenol-formaldehyde, the residual carbon framework substantially augments the density of the coating and reduces its porosity.

This comprehensive analysis delineates the critical role of the phenol-formaldehyde resin in enhancing the durability and performance of phosphate coatings under severe oxidative conditions. The observed phenomena elucidate the intricate mechanisms involved, highlighting the potential of such hybrid coatings in applications requiring high thermal stability and mechanical integrity.

**Figure 6:**
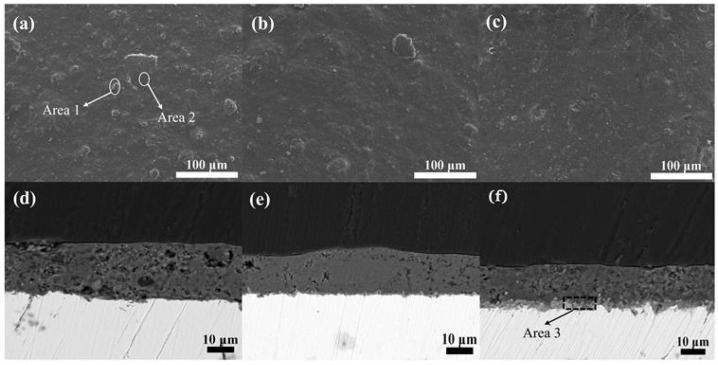
Surface and cross-sectional microstructure of phosphate coatings after high-temperature oxidation at 600 °C for 1 h, 10 h, and 100 h. (**a**,**d**) depict the surface and cross-section of the coating after 1 h at high temperature; (**b**,**e**) depict the surface and cross-section of the coating after 10 h at high temperature; (**c**,**f**) depict the surface and cross-section of the coating after 100 h at high temperature.

Consistent with top-tier scientific publication expectations, the surface and cross-sectional morphological features of hybrid phenol-formaldehyde resin phosphate coatings subjected to oxidative conditions at 600 °C for durations of 1 h, 10 h, and 100 h, as depicted in Figure 6, were meticulously characterized. Overall, the coatings subjected to oxidative conditions at 600 °C exhibit markedly superior structural and morphological integrity compared to those at 300 °C. The porosity and compactness of the coatings at various durations of oxidation at 600 °C were significantly enhanced relative to their counterparts at 300 °C, attributed to the elevated temperature facilitating a more complete carbonization of the phenol-formaldehyde resin, with only a minimal fraction of the carbonized product escaping as carbon dioxide, thus minimally affecting the internal porosity of the coating. Moreover, the coating transitioned into a vitreous state.

Figure 6a,d reveal that, despite the short duration of high-temperature oxidation, slight protuberances are visible on the coating surface, indicative of the residual carbonized phenol-formaldehyde resin. A comparative analysis of areas 1 and 2 highlights these protuberances as remnants of the carbonization process, underscoring that, although the phenol-formaldehyde resin is not inherently high-temperature resistant, its high char yield post-carbonization lends structural support to the phosphate coating, preventing fracture and contributing to toughening. Furthermore, Figure 6b,e indicate that no significant oxide film was observed between the coating and the metal substrate after 10 h of high-temperature oxidation, suggesting a further increase in the compactness of the coating. The degree of carbonization of phenol-formaldehyde and the reduction in porosity was thereby elevated, nearly satisfying the operational requirements. However, after 100 h of oxidation, an increase in the porosity was observed, as shown in Figure 6c,f. Area 3 illustrates the formation of an oxide film, owing to the reaction between the metal substrate and atmospheric oxygen, which was facilitated by the loosened and porous nature of the coating. Prolonged exposure to high temperatures led to the partial decomposition of phenol-formaldehyde.

Hence, under the conditions of 600 °C and 10 h of high-temperature oxidation, the coating achieved its optimum compactness and carbonization rate of phenol-formaldehyde. This detailed exploration elucidates the robustness and adaptability of the hybrid coatings at elevated temperatures and highlights their potential in applications requiring high thermal resistance and mechanical resilience.

**Figure 7:**
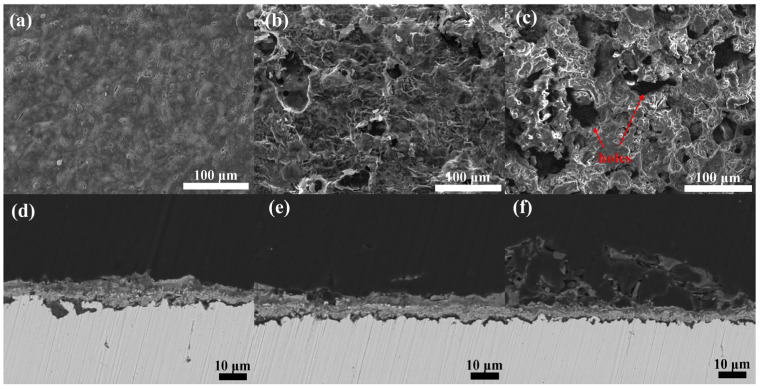
Surface and cross-sectional microstructure of phosphate coatings after high-temperature oxidation at 900 °C for 1 h, 10 h, and 100 h. (**a**,**d**) depict the surface and cross-section of the coating after 1 h at high temperature; (**b**,**e**) depict the surface and cross-section of the coating after 10 h at high temperature; (**c**,**f**) depict the surface and cross-section of the coating after 100 h at high temperature.

To satisfy the stringent criteria of discourse and precision mandated for publication in a premier scientific journal, this study conducts an exhaustive investigation of the morphological characteristics of phosphate coatings that have been hybridized with 10% phenol-formaldehyde resin. These coatings were then subjected to oxidative conditions at a temperature of 900 °C over durations of 1 h, 10 h, and 100 h. The intricate findings from this investigation are meticulously delineated in Figure 7. The coatings subjected to analysis at temperatures of 600 °C and 300 °C were found to demonstrate a contrasting level of deterioration when compared to those examined after each oxidative period at 900 °C. At this elevated temperature, the coatings displayed a spectrum of degradation levels, where the severity of corrosive damage was observed to intensify progressively with an increase in the duration of oxidation exposure. This phenomenon underscores the critical influence of temperature on the oxidative stability and longevity of the coatings, highlighting the enhanced susceptibility to degradation as the operational temperature reaches 900 °C. Despite fully transitioning into a vitreous state, the hybridized phenol-formaldehyde resin predominantly dissipated as carbon dioxide owing to the increased temperature. Additionally, elevated temperatures led to the complete dehydration of the Si-O bond structures within the coating, consequently increasing its porosity and compromising its structural integrity.

Remarkably, even after 10 h and 100 h of oxidation, parts of the carbon framework were retained, stretching around the corroded areas of the coating to preserve its fundamental structure. As seen in Figure 7a,b, the surface did not exhibit significant porosity owing to the shorter duration of oxidative corrosion. In contrast, Figure 7c,d reveal the emergence of pores on the surface after 10 h of high-temperature corrosion, likely owing to the dehydration of the Si-O bond structures compounded by the decomposition of the phenol-formaldehyde resin, leading to an increase in porosity. Figure 7e,f demonstrate that after 100 h of oxidative corrosion, the coating maintained its basic structure. The residual carbon framework supports the surrounding coating, preventing significant cracking and effectively enhancing its toughness. Cross-sectional analysis revealed the formation of microcracks between the coating and metal substrate, indicating a considerable reduction in adhesion.

This detailed investigation elucidates the resilience and degradation mechanisms of the hybrid coatings at elevated temperatures and highlights the critical role of the residual carbon framework in maintaining structural integrity under severe oxidative conditions. These findings provide the foundation for future developments in high-temperature protective coatings and emphasize the importance of optimizing the composition and processing conditions to achieve enhanced thermal stability and mechanical properties.

### 3.5. Calculation and Expression of Porosity

**Figure 8:**
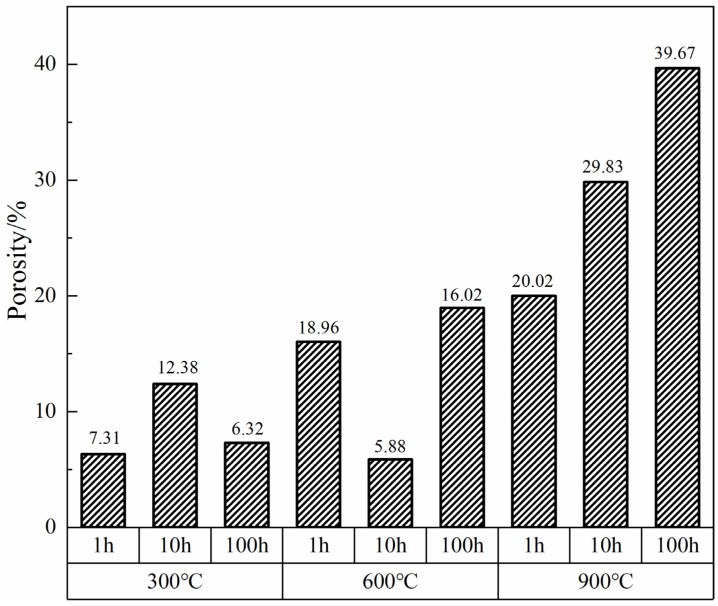
Porosity of phosphate coating at different temperatures and times after high-temperature oxidation.

Odhiambo introduced a method for calculating the porosity of coatings: by analyzing the grayscale of images using a scanning electron microscope to calculate the porosity [24]. The porosity of coatings subjected to oxidative corrosion at 300 °C, 600 °C, and 900 °C for 1 h, 10 h, and 100 h was assessed. This analysis facilitated determining the optimal service time and temperature for the coatings under high-temperature conditions and identifying the conditions under which the phenol-formaldehyde resin exhibited the highest char yield. According to Figure 8, the coatings exposed to 300 °C and subjected to 100 h of oxidative corrosion exhibited the lowest porosity of 6.32%, suggesting that the highest carbonization rate of the phenol-formaldehyde resin occurs within this duration, while the significantly higher porosity observed after 10 h of exposure implies the predominant volatilization of the resin as carbon dioxide, consistent with the morphological characteristics analyzed using SEM.

At 600 °C and after 10 h of high-temperature corrosion, the porosity further decreases to 5.88%, representing the lowest porosity among the various temperatures and durations studied. At the same time, the coating transitions into a vitreous state and the carbonization rate of the phenol-formaldehyde resin peaks, consequently maximizing the density and toughness of the coating, indicating that the coating achieved optimal protective performance under these conditions. Conversely, at 900 °C, the porosity of the hybrid coating significantly increases, as corroborated by SEM analyses showing a considerable augmentation in pore formation. Moreover, porosity escalates with the duration of oxidative corrosion, underscoring the diminished service performance of the coating at this elevated temperature compared to the outcomes at 600 °C.

Hence, the phenol-formaldehyde hybridized phosphate coating achieves its lowest porosity and, consequently, its optimal protective efficacy, under conditions of 600 °C with 10 h of oxidative corrosion, thus highlighting the importance of optimizing service conditions to enhance the protective performance of high-temperature coatings.

### 3.6. Oxidation Kinetics

**Figure 9:**
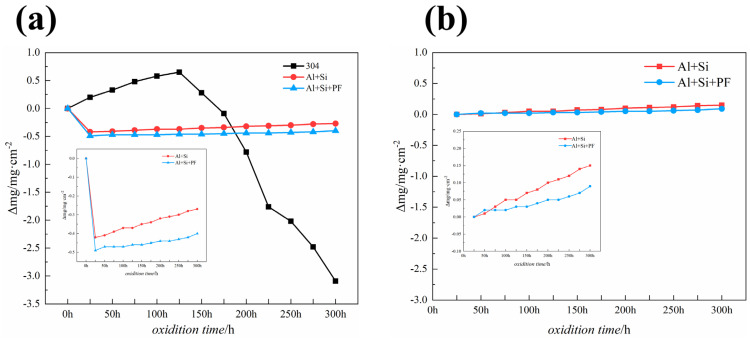
Dynamics curves of cyclic oxidation of Al+Si, Al+Si+PF, and 304 substrate at 300 °C for 300 h. (**a**) Cyclic oxidation kinetics curve from 0 to 300 h, and (**b**) cyclic oxidation kinetics curve from 25 to 300 h.

Figure 9 illustrates the mass change per unit area over time for bare 304 stainless steel, phosphate varnish-coated, and phenol-formaldehyde hybridized phosphate-coated samples subjected to cyclic oxidation at 300 °C for 300 h. As seen in Figure 9a, the 304 stainless steel exhibited a parabolic weight change behavior during the initial 125 h of cyclic oxidation. However, beyond 125 h, a notable weight loss occurs, culminating in a final mass reduction of −0.39 mg cm^−2^ for the stainless steel under 300 °C cyclic oxidation for 300 h. Both the phosphate varnish and phenol-formaldehyde hybridized phosphate coatings demonstrated superior protective performance. Incorporating phenol-formaldehyde, which undergoes carbonization at 300 °C, results in a higher char yield, thereby enhancing the coating’s density and improving its protective efficacy.

Notably, both the varnish and hybridized coatings exhibited significant weight loss within the first 25 h, attributed to the volatilization of additives, water, and part of the phenol-formaldehyde resin transformed into gas. Therefore, calculating the weight loss rate after the first 25 h or upon completion of the initial oxidation cycle yields a more accurate measure of performance. According to Figure 9b, under cyclic oxidation at 300 °C, the weight loss rates for the phosphate varnish coating and phenol-formaldehyde hybridized phosphate coating are 0.15 mg cm^−2^ and 0.09 mg cm^−2^, respectively. These data underscore the enhanced protective capability of the hybrid coating, attributed to the densification effect and char residue of the phenol-formaldehyde resin, which offers superior resistance against high-temperature oxidation.

**Figure 10:**
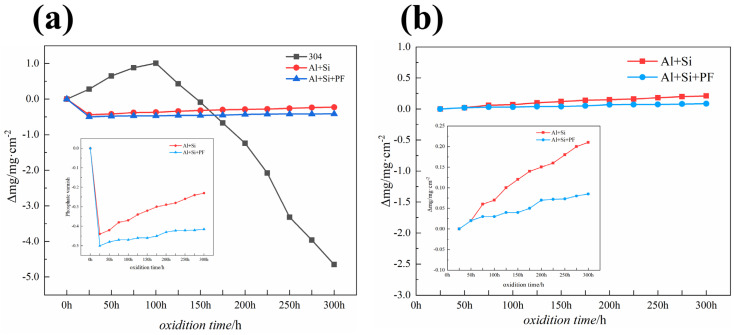
Dynamics curves of cyclic oxidation of Al+Si, Al+Si+PF, and 304 substrate at 600 °C for 300 h. (**a**) Cyclic oxidation kinetics curve from 0 to 300 h, and (**b**) cyclic oxidation kinetics curve from 25 to 300 h.

Figure 10a delineates the mass change per unit area over time following 300 h of cyclic oxidation at 600 °C, exhibiting a trend broadly similar to that observed during cyclic oxidation at 300 °C. The pure 304 stainless steel substrate demonstrated an initial mass increase within the first 100 h, adhering to the parabolic law of oxidation weight gain, but experienced a marked mass reduction over the subsequent 200 h. This weight loss can be attributed to the volatilization of certain metal oxides under prolonged exposure to high-temperature (600 °C) oxidative conditions, leading to a decrease in the mass of the metal substrate, with the 304 stainless steel substrate ultimately losing −4.65 mg cm^−2^.

The phosphate varnish and phenol-formaldehyde resin-hybridized phosphate coatings exhibited exceptional protective performances at this temperature. Both the varnish and hybrid coatings exhibited notable weight loss within the first 25 h, primarily owing to the volatilization of organic additives, solvents, and a portion of the phenol-formaldehyde resin transforming into carbon dioxide gas, resulting in a reduction in mass. Therefore, the samples post-25 h serve as a more accurate baseline for the kinetic curves shown in Figure 10b. The phosphate varnish coating experienced a final weight loss of 0.21 mg cm^−2^ over the 25–300 h of oxidative corrosion. The phenol-formaldehyde resin hybridized phosphate coating, benefiting from the high char yield of phenol-formaldehyde at 600 °C, which further increased the coating’s density, exhibited superior protective performance, with a final weight loss of 0.085 mg cm^−2^. This result demonstrates a significant enhancement in the resistance of 304 stainless steel to cyclic oxidation owing to the phenol-formaldehyde hybridized phosphate coating, outperforming the phosphate varnish coating in terms of protective efficacy.

### 3.7. Protective Mechanisms of Phosphate Varnish and Phosphate Hybrid Coatings

**Figure 11:**
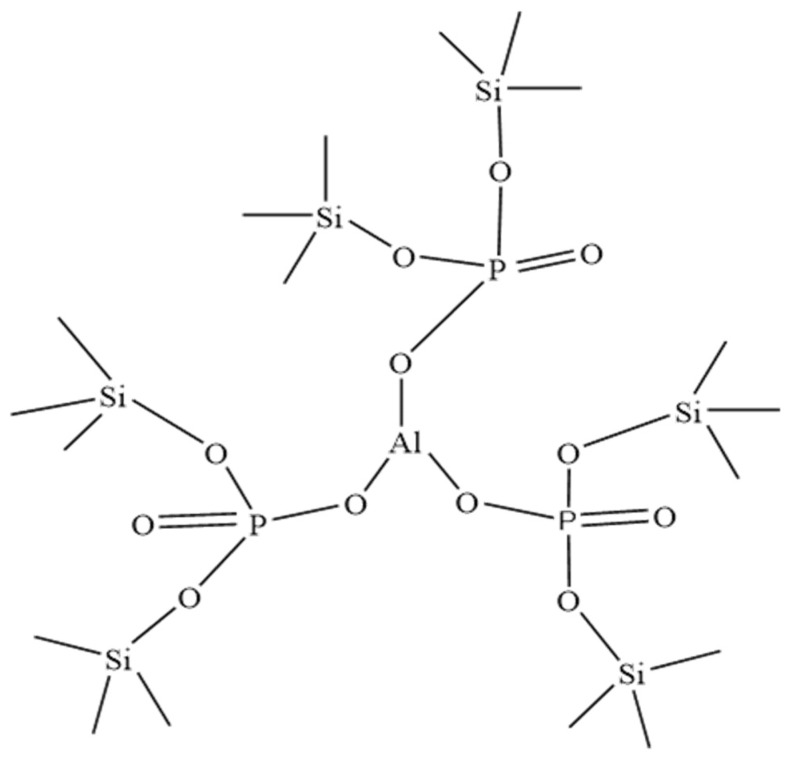
Schematic diagram of local network structure of phosphate varnish.

The protective mechanisms of phosphate varnish and phosphate hybrid coatings were comprehensively investigated. Figure 11 shows a schematic diagram of the local network structure of the phosphate varnish coating. Infrared spectroscopy analysis, as illustrated in Figure 1, indicated that the film-forming substance of the phosphate varnish was characterized by an irregular network structure formed with [-Si-O-P-] and [-Al-O-P-] as the chain segments and Al^3+^ ions serving as network-connecting nodes. These nodes of Al^3+^ are directly bonded to the oxygen atoms, which, in turn, were covalently linked to the phosphorus atoms, thereby integrating them into the network structure of the coating. This intricate network formation underpins the robust protective capabilities of phosphate varnish, highlighting the advanced materials science that facilitates the development of high-performance coatings with enhanced resistance to environmental degradation.

**Figure 12:**
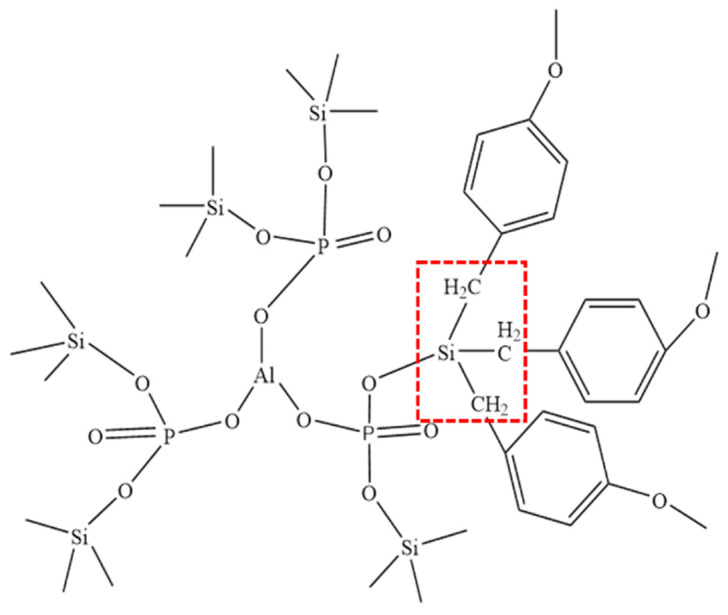
Schematic diagram of local network structure of phosphate hybrid coating.

For the phenol-formaldehyde resin-hybridized phosphate coating, XPS analysis, as depicted in Figure 2, revealed the formation of new Si-C bonds in addition to the existing [-Si-O-P-] bonds, as illustrated in Figure 12. Incorporating phenol-formaldehyde resin introduces a novel type of organic–inorganic hybrid coating characterized by these chemical bonds, which possess significant bond strength and energy. This unique chemical architecture enhances the toughness of the coating and its resistance to high-temperature oxidation [25]. This advanced material design leverages the synergy between the organic and inorganic components to achieve superior performance in protective coatings, marking a significant advancement in the development of coatings engineered to withstand harsh environmental conditions.

## 4. Conclusions

In this study, a precursor for a phenol-formaldehyde hybridized phosphate coating was synthesized using the sol-gel method, while phosphate hybrid coatings were fabricated using an air-spraying technique. Based on high-temperature oxidation corrosion tests conducted at various temperatures and durations, the hybrid coatings exhibit the lowest porosity and the highest char yield of phenol-formaldehyde resin under the conditions of 600 °C for 10 h, indicating the optimal protective performance of the coatings under these conditions. The findings are summarized as follows.

(1) Upon curing at room temperature, the coating exhibited precipitation of Al(H_2_PO_4_)_3_ crystals, at which point the protective performance of the coating was compromised. When cured at 300 °C, Al(H_2_PO_4_)_3_ undergoes further transformation into an AlPO_4_ structure. AlPO_4_ is characterized by excellent thermal stability and chemical resistance, resulting in enhanced protective properties of the coating. At temperatures of 600 °C and 900 °C, the coating transitions entirely to a vitreous state, effectively meeting service requirements.
(2) Given the inherently porous structure of phosphate coatings, reducing their porosity is a critical strategy for enhancing their protective performance. This study addresses the challenge of infilling pores with phenol-formaldehyde resin, which has a high char yield, thereby tailoring the coating to withstand high-temperature conditions. The porosity reaches its minimum (5.88%) under the conditions of 600 °C and 10 h of oxidation, indicating a considerable improvement in the coating’s ability to serve effectively under elevated temperatures.
(3) Compared to the pure 304 stainless steel substrate, the oxidative resistances of the substrates coated with phosphate varnish and phenol-formaldehyde resin-hybridized phosphate were significantly enhanced. Under the high-temperature oxidation environment of 300 °C, the weight losses for the varnish-coated and hybrid-coated substrates are 0.15 mg cm^−2^ and 0.09 mg cm^−2^, respectively. In the more severe oxidative environment of 600 °C, the varnish-coated substrate experiences a weight loss of 0.21 mg cm^−2^, while the hybrid-coated substrate shows a reduced weight loss of 0.085 mg cm^−2^. These results indicate that the phosphate coating hybridized with phenol-formaldehyde exhibited superior thermal resistance.
(4) While the phenolic resin hybridized phosphate coating has mitigated the brittleness characteristic of pure inorganic phosphate coatings, further enhancements are imperative. Although this novel hybrid coating exhibits resilience to high-temperature oxidation, its porosity requires further reduction. Incorporating partial inorganic fillers may serve as a viable solution to this challenge. Moreover, the oxidation kinetics during cyclic oxidation of this new hybrid coating present an opportunity for improvement, which could potentially be achieved through the incorporation of rare earth elements such as Y_2_O_3_ and CeO_2_. This approach aims to optimize the coating’s performance under oxidative stress, enhancing its applicability in high-temperature environments.

## Figures and Tables

**Table 1:** Chemical Composition of 304 stainless steel (wt.%).

Element	Fe	Cr	Ni	C	Mn	P	S	Si
wt.%	70.53	18	10.3	0.045	1.52	<0.0045	<0.003	0.295

**Table 2:** Other chemical agents used in the experiment.

Name	Molecular Formula	Grade	Manufacturer
Aluminum dihydrogen phosphate	Al(H_2_PO_4_)_3_	Industrial grade	Henan Zhongfan Dongsheng New Materials (Zhoukou, China)
Silica sol (SC101)	mSiO_2_·nH_2_O	Industrial grade	Henan Zhongfan Dongsheng New Materials
PF-Z819	PF	Industrial grade	Henan Zhongfan Dongsheng New Materials
Acetone	C_3_H_6_O	AR	Sinopharm Group Co., Ltd. (Shanghai, China)
Anhydrous ethanol	C_2_H_6_O	AR	Sinopharm Group Co., Ltd.
Defoaming agent	BYK-022	Industrial grade	BYK-CHEMIE (Wesel, Germany)
Surface wetting additive	FS-50	Industrial grade	Chemours Company (Wilmington, DE, USA)
Deionized water	H_2_O	RO	Prepared in-house in the laboratory

**Table 3:** Element ratios of Point 1.

Main Elements	wt.%	at.%
C	38.46%	56.16%
O	23.10%	25.32%
Al	2.22%	1.45%
Si	12.82%	8.01%
P	13.72%	7.77%

**Table 4:** Element ratio of Point 2.

Main Elements	wt.%	at.%
C	10.76%	19.31%
O	38.51%	51.90%
Al	1.96%	1.56%
Si	24.42%	18.74%
P	8.35%	5.81%

## Data Availability

The data presented in this study are available on request from the corresponding author. The data are not publicly available due to privacy restrictions.
